# Dynamics of transcriptome and chromatin accessibility revealed sequential regulation of potential transcription factors during the brown adipose tissue whitening in rabbits

**DOI:** 10.3389/fcell.2022.981661

**Published:** 2022-09-26

**Authors:** Kun Du, Guan-He Chen, Xue Bai, Li Chen, Shen-Qiang Hu, Yan-Hong Li, Guo-Ze Wang, Jing-Wei He, Song-Jia Lai

**Affiliations:** ^1^ Farm Animal Genetic Resources Exploration and Innovation Key Laboratory of Sichuan Province, Sichuan Agricultural University, Chengdu, China; ^2^ School of Public Health, The Key Laboratory of Environmental Pollution Monitoring and Disease Control, Ministry of Education, Guizhou Medical University, Guiyang, China; ^3^ Sichuan Animal Husbandry Station, Chengdu, China

**Keywords:** BAT, rabbits, ATAC-seq, epigenetics, whitening

## Abstract

Brown adipose tissue (BAT) represents a valuable target for treating obesity in humans. BAT losses of thermogenic capacity and gains a “white adipose tissue-like (WAT-like)” phenotype (BAT whitening) under thermoneutral environments, which could lead to potential low therapy responsiveness in BAT-based obesity treatments. However, the epigenetic mechanisms of BAT whitening remain largely unknown. In this study, BATs were collected from rabbits at day0 (D0), D15, D85, and 2 years (Y2). RNA-sequencing (RNA-seq) and the assay for transposase-accessible chromatin with high-throughput sequencing (ATAC-seq) were performed to investigate transcriptome and chromatin accessibility of BATs at the four whitening stages, respectively. Our data showed that many genes and chromatin accessible regions (refer to as “peaks”) were identified as significantly changed during BAT whitening in rabbits. The BAT-selective genes downregulated while WAT-selective genes upregulated from D0 to Y2, and the *de novo* lipogenesis-related genes reached the highest expression levels at D85. Both the highly expressed genes and accessible regions in Y2 were significantly enriched in immune response-related signal pathways. Analysis of different relationships between peaks and their nearby genes found an increased proportion of the synchronous changes between chromatin accessibility and gene expression during BAT whitening. The synergistic changes between the chromatin accessibility of promoter and the gene expression were found in the key adipose genes. The upregulated genes which contained increased peaks were significantly enriched in the PI3K-Akt signaling pathway, steroid biosynthesis, TGF-beta signaling pathway, osteoclast differentiation, and dilated cardiomyopathy. Moreover, the footprinting analysis suggested that sequential regulation of potential transcription factors (TFs) mediated the loss of thermogenic phenotype and the gain of a WAT-like phenotype of BAT. In conclusion, our study provided the transcriptional and epigenetic frameworks for understanding BAT whitening in rabbits for the first time and might facilitate potential insights into BAT-based obesity treatments.

## Introduction

Brown adipose tissue (BAT) represents a valuable target for treating obesity in humans (Cohen et al., 2014; Zheng et al., 2017). BAT can be found in the interscapular region of most newborn mammals, which is also known as classical BAT and is characterized by containing small and multilocular adipocytes with highly expressed uncoupling protein 1 (UCP1) (Lidell et al., 2013). Except in hibernating animals and some rodents, the classical BAT was barely detectable in adults because it undergoes conversion to a “white adipose tissue-like” phenotype (as known as “BAT whitening”) with age, which was along with functional loss of energy expenditure capacity, increased lipid accumulation, and decreased mitochondrial mass (de Jong et al., 2019; Škop et al., 2020). Although prolonged cold exposure can recover the thermogenic capacity of classical BAT and recruit “BAT-like” fat in white adipose tissue (WAT) depots (the latter was known as “beige fat”), the subjects often spend most of their lives under thermoneutral environments, which were prone to promote the BAT whitening (Lim et al., 2012; Bai et al., 2017; Škop et al., 2020; Li et al., 2022). The BAT whitening could lead to potential low therapy responsiveness in BAT-based obesity treatments (Schlein et al., 2021). Understanding the mechanism underlying BAT whitening is thus an area of immense interest.

Previous study has revealed that BAT whitening could lead to brown adipocyte death and adipose tissue inflammation (Kotzbeck et al., 2018), and many biological processes were found to be involved in the BAT whitening, such as vascular rarefaction (Shimizu et al., 2014), mitophagy (Altshuler-Keylin et al., 2016), and endogenous *de novo* lipid (DNL) synthesis (Schlein et al., 2021). The increased mitochondrial calcium level, as well as deficiencies of the leptin receptor, beta-adrenergic signaling, and lipase, were found to cause the BAT whitening (Kotzbeck et al., 2018; Gao et al., 2020). While several dietary factors, such as β-hydroxy-β-methylbutyrate, folic acid, and *Dunaliella Tertiolecta*, were identified to counter BAT whitening and decrease lipid accumulation (Duan et al., 2019; Gao et al., 2020; Yamashita et al., 2020; Chen et al., 2022). At the molecular level, the carbohydrate-response element-binding protein (ChREBP) (Schlein et al., 2021) and Reticulon-4 (Nogo) (Wang et al., 2022) were identified to promote while a BAT-selective TF, PPARA (Miranda et al., 2020), was found to inhibit BAT whitening. Although the exciting progresses in recent years, very litter is known about epigenetic mechanisms underlying BAT whitening.

Rabbits retain a high proportion of classical BAT (ca. 5% of total body) and have long been an ideal model for investigating BAT development and functions (Hardman et al., 1971; Harris et al., 1986; Comeglio et al., 2018). The abundant BAT might be a factor that keeps rabbits lean and enables rabbits to yield low-fat meats (Abdelatty et al., 2021). Naturally, the classical BAT of rabbits losses its thermogenic phenotype during the growth period (Derry et al., 1972). Given the large size and easy accessibility of infant BAT, rabbits exhibit great importance in revealing the whitening process of BAT. Our previous study characterized the morphology changes during BAT whitening in rabbits by using H&E staining and immunohistochemistry and found increased lipid accumulation and decreased proportion of classical brown adipocytes during BAT whitening (Du et al., 2021). In this study, we performed RNA-sequencing (RNA-seq) and the assay for transposase-accessible chromatin with high-throughput sequencing (ATAC-seq) to investigate the dynamics and functions of the transcriptome and chromatin accessibility at four growth stages of rabbits. Then, a combined analysis of RNA-seq data and ATAC-seq data identified the relationship between chromatin accessibility and gene expression of BAT. Finally, TF footprinting analysis revealed sequential regulation of TF during BAT whitening. Our work is the first report on the genome-wide chromatin accessibility of BATs in rabbits and provides an important resource for further BAT-based studies in fighting against obesity.

## Materials and methods

### Ethics statement

This study was performed in accordance with the guidelines of Good Experimental Practices adopted by the Institute of Animal Science (Sichuan Agricultural University, Chengdu, China). All surgical procedures involving rabbits were performed according to approved protocols of the Biological Studies Animal Care and Use Committee, Sichuan Province, China.

### Tissue sample preparation

In this study, the Tianfu Black rabbits (native species in Sichuan province of China) were raised at the breeding center of Sichuan Agricultural University, Ya’an, China. These rabbits were breast-fed before 30 days and then fed with the same standard diet (a commercial pelleted food (16% protein, 10.8 MJ/kg)) as described in our previous study after weaning (Wang G. et al., 2020) and water *ad libitum*. Interscapular adipose tissues were collected from 12 male rabbits at different growth stages of 0 days (D0), D15, D85, and 2 years (Y2) (three individuals per stage), which represent the infant stage, early whitening stage, puberty stage, and elder stage, respectively. These adipose tissues were immediately isolated from rabbits under sterile conditions, snap-frozen in liquid nitrogen, and stored at –80°C until RNA extraction or cell isolation.

### Strand-specific RNA-sequencing and data processing

The strand-specific RNA-sequencing (ssRNA-seq) was performed as described in our previous study (Wang et al., 2018). Briefly, after RNA extraction and quality check, approximately 1 μg total RNA undergone rRNA removing and ssRNA-seq libraries construction using TruSeq Stranded Total RNA Library Prep Gold (TruSeq, San Diego, California, United States) according to the manufacturer’s recommendation. Then the purified libraries were sequenced on an Illumina NovaSeq 6,000 platform and 150 bp orientational paired-end reads were generated. The quality of ssRNA-seq reads was ascertained through Fastqc program (v0.11.8) (Brown et al., 2017). Sequencing adapters and low-quality reads were removed using Cutadapt (v3.2) software (Martin, 2011) with parameters of ‘-e 0.1 -a AGA​TCG​GAA​GAG​CAC​ACG​TCT​GAA​CTC​CAG​TCA​C -A AGA​TCG​GAA​GAG​CGT​CGT​GTA​GGG​AAA​GAG​TGT -m 100 --cut 0 -O 13’. Clean reads were mapped back to the rabbit reference genome (OryCun2.0, Ensembl release 101) using HISAT2 alignment tool (v2.1.0) (Kim et al., 2015) with default parameters. The stringtie ‘-eB’ and ‘-A’ were used to estimate raw read counts and gene expression abundance using the method of transcripts per kilobase of exon model per million mapped reads (TPM), respectively. Differentially expressed genes (DEGs) were identified using DESeq2 (Love et al., 2014) and the *p*-values of hypothetical tests were adjusted using the method of false discovery rate (FDR). The genes with the thresholds of | log_2_ (fold-change) | > 1 and FDR <0.01 were considered DEGs.

### ATAC-sequencing and data processing

Approximately 50,000 cells of eachsample were used for ATAC-sequencing (ATAC-seq). The ATAC-seq libraries were constructed using TruePrep DNA Library Prep Kit V2 for Illumina (Vazyme Biotech Co., Ltd Nanjing, China) according to the manufacturer’s recommendation. After chromatin was extracted from cells, Tn5 transposase was applied for processing DNA fragmentation and adapter incorporation. The quality of the ATAC-seq libraries was assessed using Agilent 2,100 (Agilent Technologies, Carlsbad, CA, United States) and all the libraries were sequenced on an Illumina NovaSeq 6,000 platform. The sequencing quality check of ATAC-seq was performed using Fastqc (Brown et al., 2017). Sequencing adapters and low-quality reads were removed using Cutadapt program (Martin, 2011) with the parameters of ‘-e 0.1 -a AGA​TCG​GAA​GAG​CAC​ACG​TCT​GAA​CTC​CAG​TCA​C -A AGA​TCG​GAA​GAG​CGT​CGT​GTA​GGG​AAA​GAG​TGT -m 100 --cut 0 -O 3’. Clean reads were mapped back to the rabbit genome using Bowtie2 (v2.4.1) software with the parameter of ‘-X 1000’ (Langmead and Salzberg, 2012). Then, SAMtools (v1.7) (Li et al., 2009) was used to remove failed mapping reads, mitochondrial DNA mapping reads, and low mapping score (MAPQ score <20) reads. The PCR duplicates were marked and removed using the MarkDuplicates program in Picard (v2.24.1) (Tarasov et al., 2015). Masc2 (2.2.7.1) (Zhang et al., 2008) was used to call chromatin accessible regions (as known as ‘ATAC peaks’) for each sample with parameters of ‘--nomodel --shift -100 --extsize 200’ and *q* value cutoff for peak calling was 0.05. CHIPSeeker (Yu et al., 2015) was used to annotate genome-wide ATAC-seq peaks. To identify differential peaks (DPs), a consensus peak set that merged all peaks from the 12 samples was generated using DiffBind (Ross-Innes et al., 2012) with the parameter of ‘MinOverlap 2’. DiffBind was also used to estimate of peak intensity (‘DBA_SCORE_READS’ in DiffBind). The peak intensity was normalized using CPM, which was calculated by dividing raw read counts by the count number of reads that mapped to all peaks. DP analysis was performed using DESeq2 (Love et al., 2014) and the peaks with the thresholds of |log_2_ (fold-change)| > 1.5 and FDR <0.01 were considered DPs. The Z-scaled CPM was used to cluster DPs using the K-means method by the R package of Complexheatmap (Gu et al., 2016). The ATAC-seq signals around gene bodies were calculated by Deeptools (Ramírez et al., 2014) and visualized by EnrichedHeatmap (Gu et al., 2018). The transcription factor (TF) binding motif enrichment was conducted using HOMER (v4.9.1) (Xie et al., 2020). The enriched motifs with *p*-value < 0.01 were considered significant.

### TF footprinting analysis

The vertebrate TF motifs were downloaded from the JASPAR database (release 2021) (Fornes et al., 2020). The TOBIAS (Bentsen et al., 2020) was employed to predict (TF) binding sites and measure footprint of TFs. Briefly, the Tn5 transposase sequence preference of cutting sites was estimated and corrected using the parameter of ‘ATACorrect’. The deletion of ATAC-seq signals given rise from protein binding and the neighboring signals around binding sites were calculated using the parameter of ‘FootprintScores’. The differential binding TFs were detected using the parameter of ‘BINDetect’. All TFs with - log10 (*p*-value) above the 95% quantile or differential binding scores smaller/larger than the 5 and 95% quantiles (top 5% in each direction) were considered differential binding TFs (DBTFs) (Bentsen et al., 2020). The protein-protein interaction was predicted using String database (Szklarczyk et al., 2021).

### Functional enrichment and pathway analysis

Gene ontology (GO) and Kyoto Encyclopedia of Genes and Genomes enrichment (KEGG) pathway analysis were conducted using clusterProfiler (Yu et al., 2012). The enriched GO terms and KEGG pathways that have a *p*-value < 0.05 were considered significant.

### Quantitative real-time PCR (qRT-PCR)

The qRT-PCR primers were designed using online software of primer-BLAST (https://www.ncbi.nlm.nih.gov/tools/primer-blast, Supplementary Table S1). Total RNA was reversed transcribed to cDNA using PrimeScripts RT Reagent Kit containing gDNA Eraser (TAKARA, Dalian, China). Then the cDNA template was used for qPCR and was determined using SYBR II master mix kit (TAKARA, Dalian, China). The qPCR was performed on a Bio-Rad CFX manager according to the manufacturer’s instructions. The amplification reaction was conducted under the following program: pre-denaturation at 95°C for 10 s and followed by 40 cycles of denaturation at 95°C for 5 s and annealing/extension at 59°C for 20 s. The melting curve analysis was performed from 55 to 95°C with an increment of 0.5°C. All the qRT-PCR Ct-values were normalized to the Ct-value of the *18S RNA* using the 2^−ΔΔCt^ method.

### Statistical analysis

Statistical analyses including *t*-test and one-way ANOVA were conducted on R software. The *p*-value < 0.05 was considered significant.

## Results

### Overview of transcriptome during BAT whitening

**FIGURE 1:**
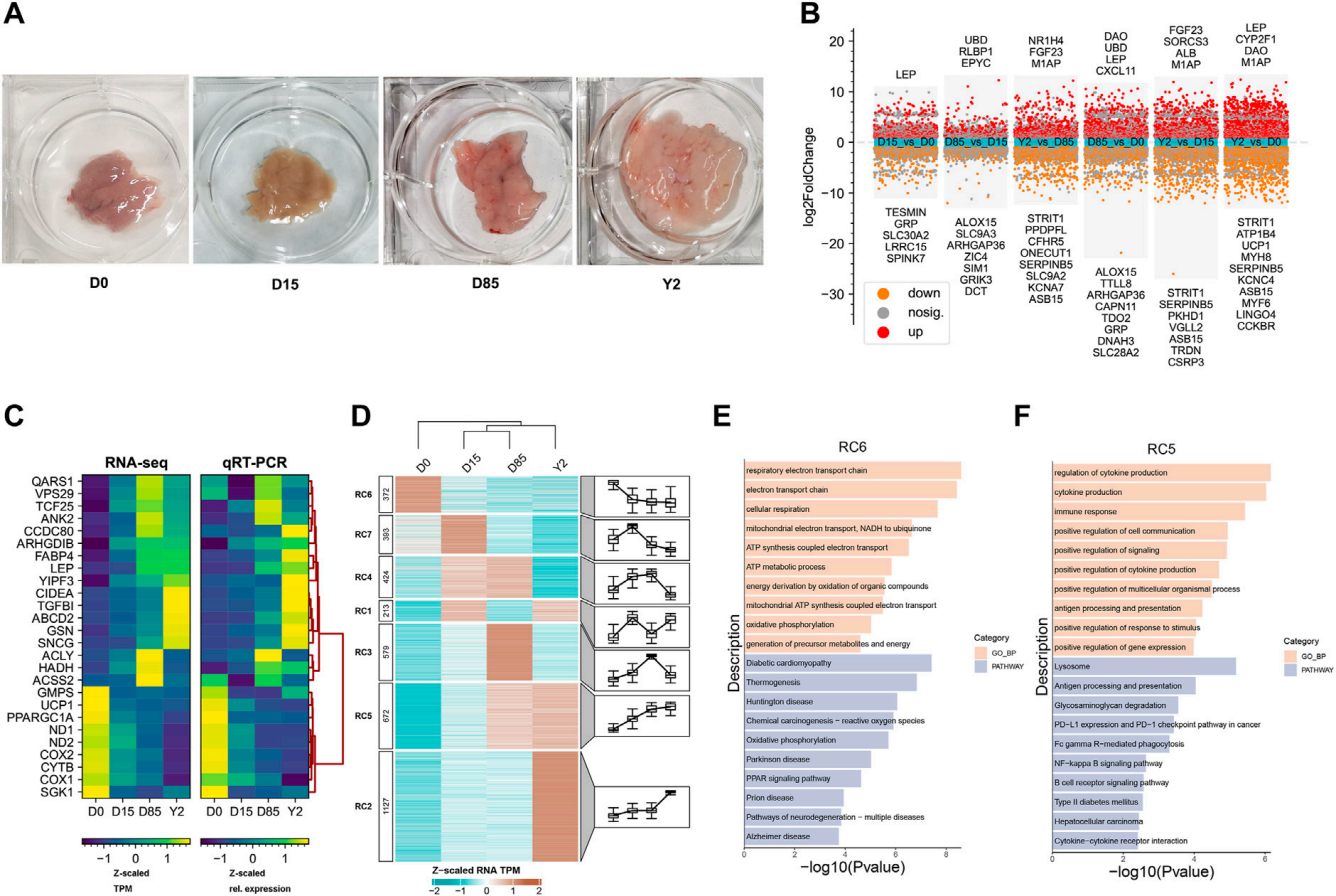
Transcriptome analysis of BAT whitening in rabbits. **(A)** The appearance of BATs at four stages. **(B)** Differential analysis of the transcriptome. The scatter plot show the genes with log2(FC) > 1 or log2(FC) < - 1. The red and orange points show the genes with FDR <0.01. The grey points show the genes with FDR ≥0.01. The top 10 up- and downregulated DEGs that had been assigned official gene symbols were shown in corresponding directions. **(C)** qRT-PCR validation of selected genes in RNA-seq. The Ct-values of qRT-PCR are normalized to D0 and *RN18S*. Three independent samples are set per group and two technical replicates are set for one individual experimental replicate in qRT-PCR. **(D)** Heatmap analysis of differentially expressed genes (DEGs) based on the K-means clustering method. TPM values of genes are Z-scaled by row. The expression patterns of DEGs in different clusters are shown in corresponding box-plots. The numbers of genes in different clusters are presented in the left annotation of the heatmap. **(E,F)** Bar plots show the top 10 enriched Gene Ontology enrichment in biological process category (GO-BP) terms and Kyoto Encyclopedia of Genes and Genomes (KEGG) pathways of genes in RC6 and RC2, respectively. Landscape and dynamics of chromatin accessibility during BAT whitening.

To investigate the BAT whitening, we collected BATs from interscapular regions of growing rabbits (*n* = 3 per growth stage), including that in the infant stage (0 days, D0), early thermogenic adaptation stage (D15), puberty stage (D85), and elder stage (2 years, Y2). Appearances of BAT showed that the brown color gradually faded from D0 to Y2 and morphology of Y2 samples was similar to visceral WAT (Figure 1A, data not shown for visceral WAT). RNA-seq found that many known BAT-selective genes dramatically downregulated while some known WAT-selective genes upregulated during BAT whitening. For instance, the BAT-selective genes *UCP1*, *COX1*, and *PPARGC1A* were dramatically downregulated from D0 to D85 and were almost not expressed in Y2; the WAT-selective genes *LEP*, *SNCG*, and *CCDC80* were almost not expressed in D0 but were greatly upregulated from D15 to Y2. On the other hand, two lipogenesis-related genes *FABP4* and *GSN* were gradually upregulated from D0 to Y2, and two DNL-related genes *ACSS2* and *ACLY* reached the highest expression levels at D85 and then downregulated from D85 to Y2 (Supplementary Figure S1A; Supplementary Table S2). Unexpectedly, one BAT-selective gene, *CIDEA*, was found to be upregulated from D0 to Y2.

Paired comparison analyses identified 890, 1,242, 2,267, 277, 1,053, and 947 upregulated differentially expressed genes (DEGs) and 574, 909, 1,653, 264, 1,047, and 1,042 downregulated DEGs in D15 *vs.* D0, D85 *vs.* D0, Y2 *vs.* D0, D85 *vs.* D15, Y2 *vs.* D15, and Y2 *vs.* D85, respectively (Figure 1B). The RNA-seq data were validated using qRT-PCR. A total of 26 genes were selected to be validated, including nine known BAT-selective genes (*UCP1*, *PPARGC1A*, *CIDEA*, *HADH*, *ND1*, *ND2*, *COX2*, *CYTB* and *COX1*), four WAT-selective genes (*LEP*, *SNCG*, *ABCD2*, and *CCDC80*), two lipogenesis genes (*FABP4* and *GSN*), two DNL genes (*ASCCL2* and *ACLY*), and nine randomly selected genes (*QARS1*, *VPS29*, *TCF25*, *ANK2*, *ARHGDIB*, *TGFBI*, *YIPF3*, *GMPS*, and *SGK1*). The qRT-PCR validation showed that the patterns of expression changes of selected genes were similar to that detected in the RNA-seq, indicating the reliable RNA-seq data (Figure 1C).

K-means clustering approach based on TPM value was used to sort the DEGs into seven clusters, named RC1 to RC7, respectively (Figure 1D). The genes in RC6 were downregulated from D0 to Y2. Gene Ontology enrichment in biological process category (GO-BP) and Kyoto Encyclopedia of Genes and Genomes (KEGG) pathways analysis showed that the genes in RC6 were strongly enriched in energy expenditure associated terms or signal pathways, such as respiratory electron transport chain, and electron transport chain, cellular respiration, as well as thermogenesis and oxidative phosphorylation signal pathways (Figure 1E). The genes in RC7 were temporarily expressed in D15 and then downregulated from D15 to Y2. GO enrichment revealed that RC7 contains genes involved in skeletal muscle development (Supplementary Figure S1B). The genes in RC4 were upregulated from D0 to D85 and were significantly enriched in mitochondrial development associated GO-BP terms, such as mitochondrion organization, NADH dehydrogenase complex assembly, and mitochondrial respiratory chain complex I assembly. The RC4 was also enriched in the fatty acid metabolism KEGG pathways, such as and TCA cycle, fatty acid metabolism, and thermogenesis (Supplementary Figure S1B). The genes in RC1 were highly expressed in D15 and Y2. The top three enriched GO-BP terms were tissue development, muscle system process, and muscle contraction, which were similar with that of RC7 (Supplementary Figure S1B). The genes in RC3 were upregulated from D0 to D85 and then downregulated in Y2. The RC3 was significantly enriched in the cell proliferation-related GO-BP terms, such as chromosome segregation, mitotic sister chromatid segregation, and mitotic nuclear division. KEGG pathway analysis showed RC3 was significantly enriched in cell cycle (Supplementary Figure S1B). The genes in RC5, such as IRFs (IRF1, IRF5, and IRF8), TGFB3, and TLR3, were continuously upregulated from D0 to D85 and retained high expression in Y2. RC5 was significantly enriched in immune function GO-BP terms, such as regulation of cytokine production, cytokine production, and immune response. The top3 enriched KEGG pathway by the genes in RC5 were lysosome, antigen processing and presentation, and glycosaminoglycan degradation (Figure 1F). RC2 was composed of the most DEGs (n = 1,127) and its genes upregulated from D0 to Y2. The RC2 was significantly enriched in cell proliferation, such as regulation of cell population proliferation and cell population proliferation. The top three enriched KEGG pathways by RC2 were TGF-beta signaling pathway, MAPK signaling pathway, and calcium signaling pathway (Supplementary Figure S1B).

In summary, RNA-seq data revealed the downregulation of BAT-selective genes contributed to the loss of thermogenic phenotype, and the upregulation of WAT-selective genes, lipogenesis-related genes, and DNL-related genes might promote to the formation of WAT-like phenotype of classical BAT in rabbits. Clustering analysis found that downregulated DEGs during BAT whitening were involved in thermogenic capacity, while the upregulated DEGs during BAT whitening were involved in the immune response-related functions and cell proliferation.

### Landscape and dynamics of chromatin accessibility during BAT whitening

**FIGURE 2:**
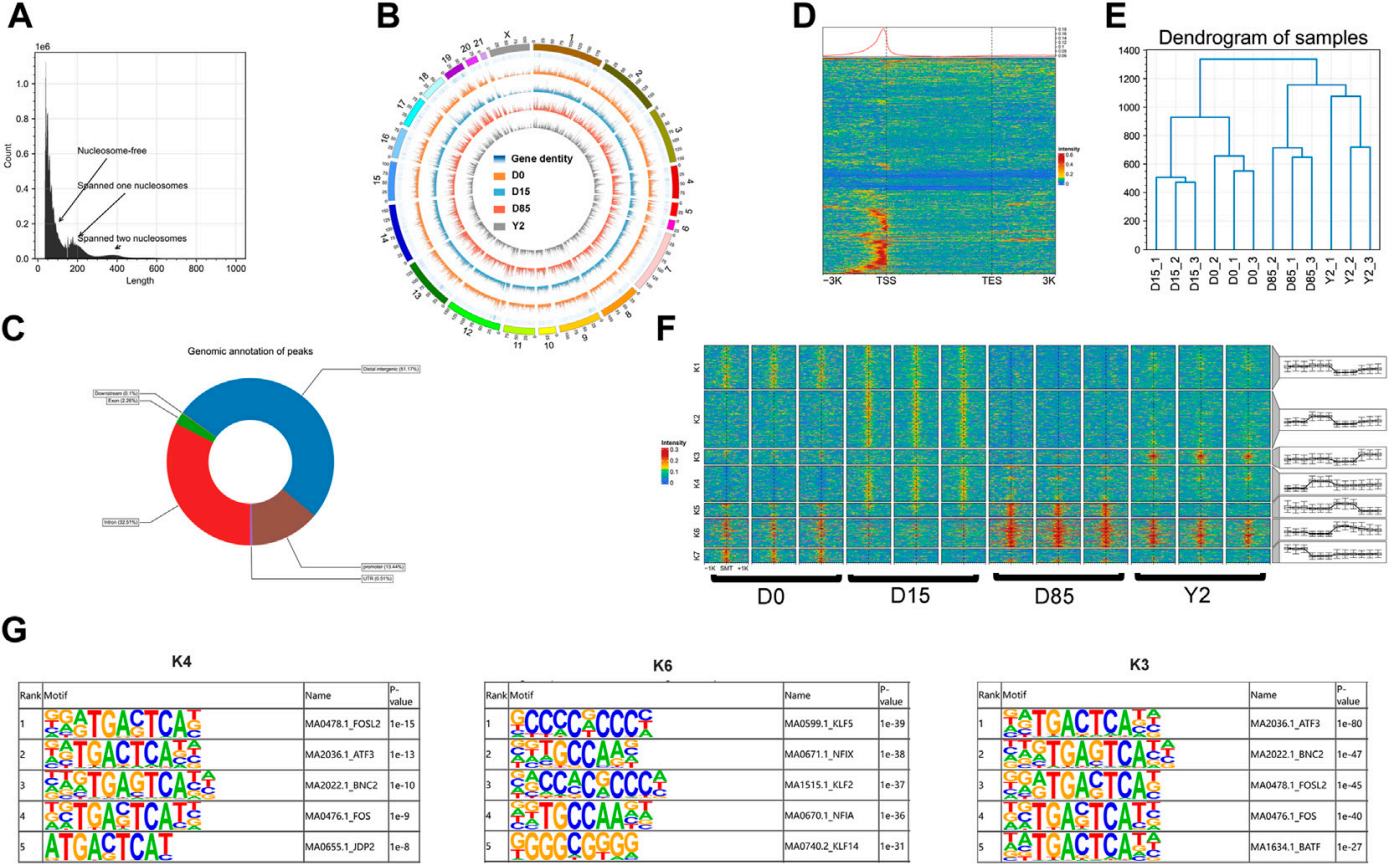
The landscape and dynamics of chromatin accessibility during rabbit BAT whitening. **(A)** Fragment lengths detected in a representative ATAC-seq library. **(B)** Genome-wide chromatin accessibility of samples in D0, D15, D85, and Y2. **(C)** Genomic annotation of peaks. **(D)** Enrichment of ATAC-seq reads around the gene bodies of a representative sample. The red and blue colors show the higher and lower ATAC-seq signals, respectively. **(E)** Hierarchical clustering analysis based on the signal intensity of genome-wide chromatin accessible regions (peaks). The signal intensities of peaks were normalized using CPM. **(F)** Heatmap analysis of 25,464 differential peaks (DPs) using K-means method. The color scale shows the relative signal intensities of peaks. SMT, the summit of peaks. The chromatin accessible patterns of DPs in different clusters of each replicate are shown in corresponding box-plots. **(G)** TF binding motif enrichments of peaks in cluster K4, K6, and K3. Integrated analysis of ATAC-seq and RNA-seq data revealed chromatin accessibility regulating gene expression.

To determine the global epigenetic landscape during whitening process of the BAT, we employed ATAC-seq to interrogate chromatin accessibility of samples (n = 3 per stage). We obtained an average of 250.00 million qualified reads per sample (Supplementary Table S3). The Q20 ratio, Q30 ratio, total read mapping rate, and uniquely read mapping ratio varied from 95.86 to 98.25%, 88.87–95.09%, 80.16–92.55%, and 55.82–64.01%, respectively (Supplementary Table S3). All ATAC-seq libraries yielded the expected length distribution of inserting fragments, with a great portion of the fragments being short, representing the intervening region between two consecutive nucleosomes, and progressively few fragments of larger length size, representing spanned one or more nucleosomes (Figure 2A). A total of 214,204 chromatin accessible regions (refer to as ‘ATAC peaks’ thereafter) were identified across all samples, which were widely distributed throughout the genome and their distribution was found to be positively related to gene density in the genome in general (Figure 2B; Supplementary Table S4). Analysis of the reads in peaks showed the values of fraction of reads in called peaks (FRiP) ranged from 0.060 to 0.124 in the samples (Supplementary Table S3). Genomic annotation of identified peaks revealed that major peaks (51.17%) were located at distal intergenic regions, and 32.51 and 13.44% of peaks were located at the intronic regions and promoter regions (±3 kb of transcriptional start sites, TSS), respectively (Figure 2C). As expected, ATAC-seq peaks were strongly enriched around the TSSs of genes (Figure 2D), which demonstrated the reliability and high quality of our ATAC-seq data. Hierarchical clustering analysis based on the peak intensity showed the high reproduction of replicate samples (Figure 2E). On the other hand, correlation analyses of the peak intensity were conducted to detect the similarities of all replicate samples and excellent reproducibility was obtained in this study (Supplementary Figure S2A). Collectively, our data provided the genome-wide chromatin landscape of BATs in rabbits for the first time.

To investigate how chromatin accessibility dynamics govern the BAT whitening, we compared signal intensities of ATAC-seq peaks for paired comparisons of four stages. A total of 5,841, 5,546, 6,522, 3,918, 4,594, and 2,777 increased peaks and 1854, 10,010, 4,272, 12,959, 2,431, and 1,690 decreased peaks were detected in D15 *vs.* D0, D85 *vs.* D0, Y2 *vs.* D0, D85 *vs.* D15, Y2 *vs.* D15, and Y2 *vs.* D85, respectively (Supplementary Figure S2B). The union set of differential peaks (DPs) from different comparisons were compose of 25,464 peaks. The obtained DPs were subjected to K-means clustering and seven distinct clusters (K1 - K7) were identified (Figure 2F). The clusters K1 - K7 were composed of 5,361, 7,017, 1804, 4,374, 1,692, 3,523, and 1,693 peaks, respectively. We then performed GO-BP enrichment and KEGG pathway analysis for the nearest expressed genes of these peaks. The K4, K6, and K3 represented the increasing chromatin accessibility from D0 to D15, D15 to D85, and D85 to Y2, respectively. The peaks in K4 exclusively closed in D0 and their accessibility dramatically increased from D0 to D15. The top three enriched GO-BP terms were potassium ion transmembrane transport, cell death in response to oxidative stress, and negative regulation of DNA-binding transcription factor activity. KEGG pathway analysis revealed that peaks in K4 were strongly enriched in the lipid metabolisms, such as thyroid hormone signaling pathway, glycosaminoglycan degradation, lysosome, and PPAR signaling pathway (Supplementary Figure S2C). The top five enriched TF binding motifs by the peaks in K4 were FOSL2, ATF3, BNC2, FOS, and JDP2 (Figure 2G). The peaks in K6 kept the moderate chromatin accessibility in D0 and D15 and increased accessibility from D15 to D85. GO-BP enrichment revealed that peaks in K6 were enriched in positive regulation of apoptotic process and circulatory system development. KEGG pathway analysis revealed that peaks in K6 were strongly enriched in lipid accumulation associated pathways, such as the top two enriched PPAR signaling pathway and adipocytokine signaling pathway, the AMPK signaling pathway, and insulin resistance (Supplementary Figure S2C). The top five enriched TF binding motifs by the K6 were KLF5, NFIX, KLF2, NFIA, and KLF14 (Figure 2G). The chromatin accessibility of peaks in K3 was stable from D0 to D85 but dramatically increased in Y2 and were resided around the genes associated with immunity function, such as CCL22, CXCR4, and BDKRB2. Both GO and KEGG revealed that peaks in K3 were enriched in the immunity function, such as cytokine-cytokine receptor interaction, viral protein interaction with cytokine and cytokine receptor, and inflammatory mediator regulation of TRP channels. On the other hand, fat development-related GO terms were also enriched, such as fatty acid transport and long-chain fatty acid transport (Supplementary Figure S2C). The top five enriched TF binding motifs by the K3 were ATF3, BNC2, FOSL2, FOS, and BATF (Figure 2G).

In summary, ATAC-seq identified 214,204 peaks and 25,464 DPs during BAT whitening. Analysis of proximal regulation of DPs found that increasing chromatin accessibility from D0 to D15, D15 to D85, and D85 to Y2 were resided at genes involved in the lipid metabolisms, lipid accumulation, and immunity function, respectively. Three sets of TF motifs were enriched by the sequences of DPs with increasing chromatin accessibility from D0 to D15, D15 to D85, and D85 to Y2, respectively.

### Integrated analysis of ATAC-seq and RNA-seq data revealed chromatin accessibility regulating gene expression

**FIGURE 3:**
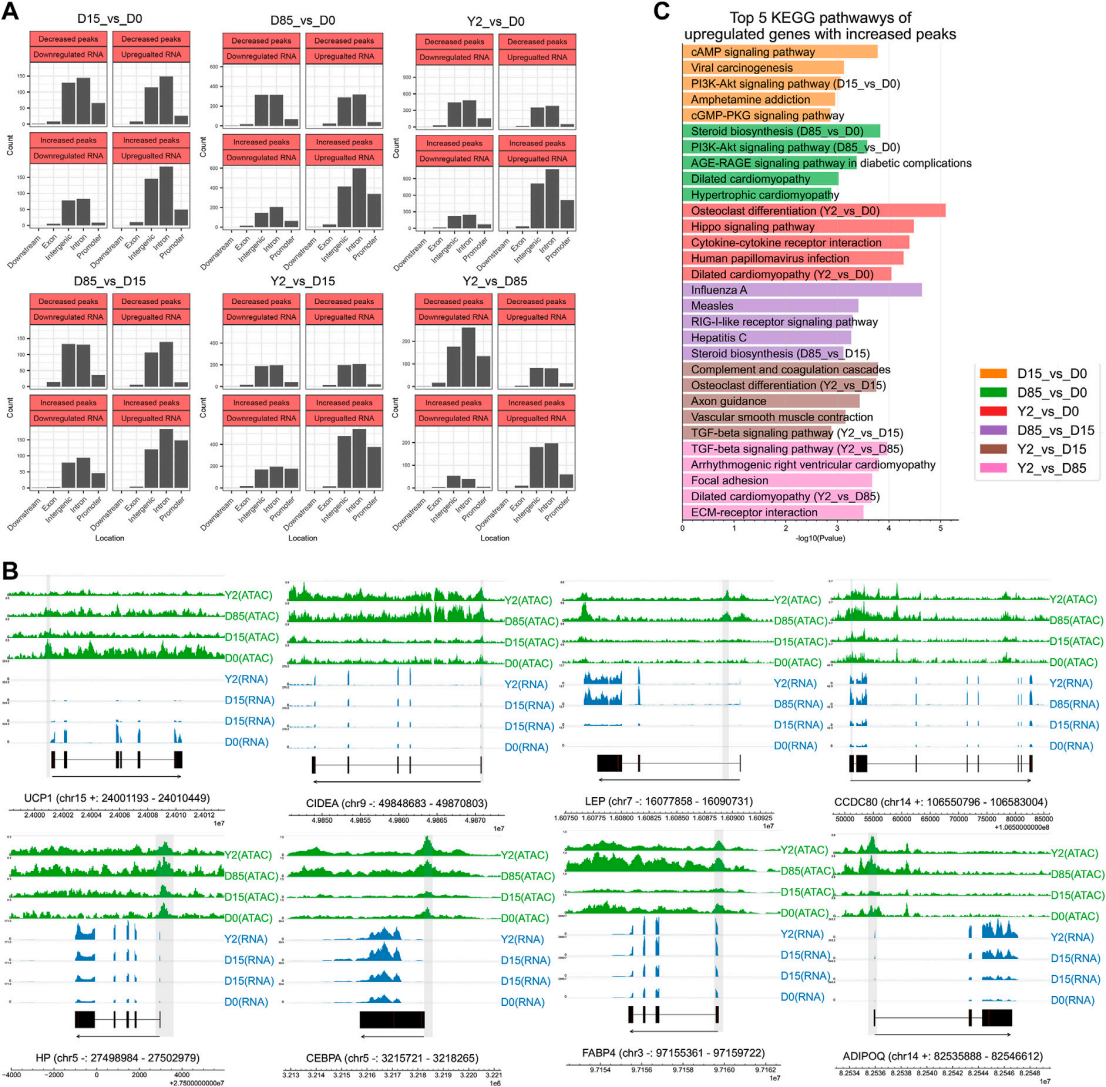
Combined analysis of ATAC-seq data and RNA-seq data. **(A)** Analysis of four types of relationship between chromatin accessibility and gene expression based on different genomic elements. **(B)** Tracks of key adipose genes. The blue and green tracks showing the reads coverage of RNA-seq and ATAC-seq. Gene exons are marked in black. Gene information is marked by “gene name (chromosome strand: start - end)”. The locations of DPs were shadowed in grey. **(C)** KEGG pathway analysis of upregulated genes with increased peaks. Genome-wide footprinting analysis revealed sequential regulation of potential TF groups.

To investigate chromatin accessibility regulating the expression of nearby genes during BAT whitening in rabbits, we performed the association analysis between DPs and DEGs based on genomic annotation of peaks. As shown in Figure 3A, a great portion of DPs were located in the distal intergenic regions and introns of DEGs. There were four types of relationships between chromatin accessibility and gene expression, including decreased peaks being located in the downregulated genes (DPDGs), decreased peaks being located in the upregulated genes (DPUGs), increased peaks being located in the downregulated genes (IPDGs), and increased peaks being located in the upregulated genes (IPUGs) (Supplementary Table S6). The DPDGs and IPUGs represented the synchronous changes of chromatin accessibility and gene expression, potentially reflecting the regulation relationship. Our results suggested remarkable changes in the proportion of the synchronous changes between chromatin accessibility and gene expression during BAT whitening. During the period from D0 to D15 and from D15 to D85, the proportion o DPDGs and IPUGs was relatively lower (60.91%, and 61.25%, respectively). While during the period from D85 to Y2, the proportion of DPDGs and IPUGs dramatically increased (78.65%). The high proportion of DPDGs and IPUGs was also found in the period from D0 to Y2 (72.35%), D15 to Y2 (64.98), and D0 to D85 (65.64%). For the DPs in promoter regions, the proportion of DPDGs and IPUGs was far more than that of DPUEs and IPDGs, including 76.67% from D0 to D15, 79.88% from D0 to D85, 84.24% from D0 to Y2, 79.84% from D15 to D85, 90.20% from D15 to Y2, and 90.65% from D85 to Y2. The high proportion of DPDGs and IPUGs of DPs in promoter regions suggested the importance of chromatin accessibility of promoter regions for regulating gene expression. Although our data showed chromatin accessibility for given genes is not always correlated with its gene expression (Supplementary Figure S2D), we found chromatin accessibility was positively correlated with the expression of some key adipose genes in general. For instance, the chromatin accessibility of the promoter region of the *UCP1*, a downregulated key thermogenic gene, was found to be decreased from D0 to Y2. While another BAT-selective gene, *CIDEA* showed both the upregulated gene expression and the increased chromatin accessibility of promoter from D0 to Y2. The chromatin accessibility of promoter regions of the *LEP*, *CCDC80*, and *HP*, three upregulated WAT-selective genes, was found to be increased from D0 to Y2. The chromatin accessibility of promoter regions at the *CEBPA*, *FABP4*, and *ADIPOQ*, three upregulated lipogenesis genes, were found to be increased from D0 to Y2 (Figure 3B). On the other hand, the chromatin accessibility of promoter regions of the *ACSS2* and *ACLY*, two DNL genes, were found to be lowly accessible at D0 and D15 and highly accessible at D85 and Y2 (Supplementary Figure S2E).

Our data showed that the number of IPUGs is more than that of DPDGs in most of studied periods (Figure 3A), which might indicate their dominant roles during BAT whitening. The KEGG pathways enriched by the DEGs of IPUGs were shown in Figure 3C. During the period from D0 to D15, the top three mostly enriched pathways were cAMP signaling pathway, viral carcinogenesis, and PI3K-Akt signaling pathway. During the period from D0 to D85, the top three mostly enriched pathways were steroid biosynthesis, PI3K-Akt signaling pathway, and AGE-RAGE signaling pathway in diabetic complications. During the period from D0 to Y2, the top three mostly enriched pathways were osteoclast differentiation, hippo signaling pathway, and cytokine-cytokine receptor interaction. During the period from D15 to D85, the top three mostly enriched pathways were influenza A, measles, and RIG-I-like receptor signaling pathway. During the period from D15 to Y2, the top three mostly enriched pathways were complementary and coagulation cascades, osteoclast differentiation, and axon guidance. During the period from D85 to Y2, the top three mostly enriched pathways were TGF-beta signaling pathway, arrhythmogenic right ventricular cardiomyopathy, and focal adhesion. The PI3K-Akt signaling pathway, steroid biosynthesis, TGF-beta signaling pathway, osteoclast differentiation, and dilated cardiomyopathy were the most frequently enriched KEGG pathways by the IPUGs in all detected periods (Figure 3C).

### Genome-wide footprinting analysis revealed sequential regulation of potential TF groups

**FIGURE 4:**
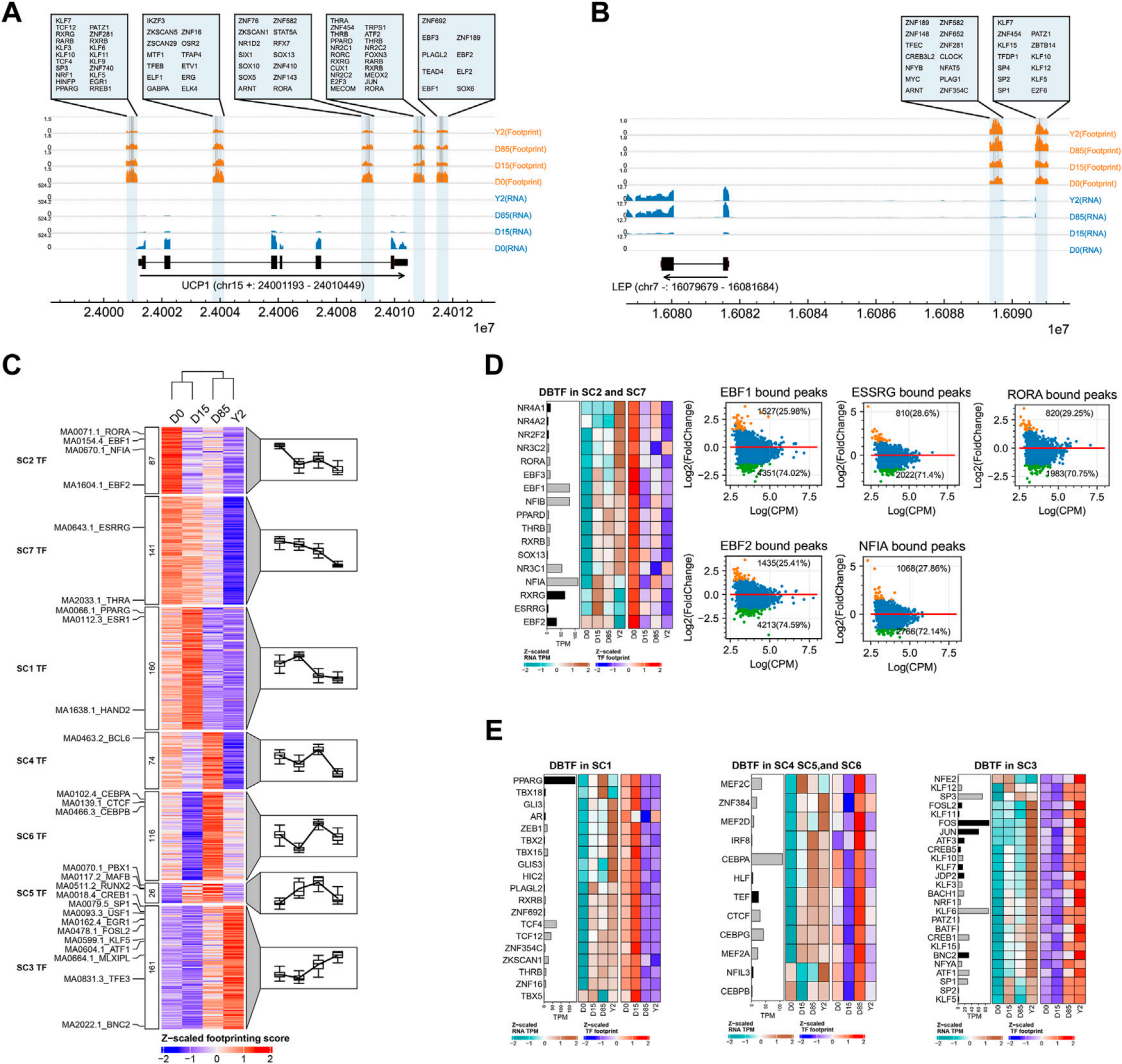
Genome-wide TF footprinting analysis during BAT whitening in rabbits. **(A,B)** The TF footprints at the loci of *UCP1* and *LEP*. The arrows showed the gene transcription directions. The blue and orange tracks showed read coverages of RNA-seq and footprint binding score of ATAC-seq. The bound TFs in highlighted regions were shown in the zoomed axes. **(C)** Clustering of TF based on footprint score. Each row represents 1 TF, and each column represents a BAT whitening stage. TF footprint binding scores are Z-scaled by row. The blue and red color indicates the low and the high footprint score, respectively. Each cluster is associated with a mean trend line (left to right) and timepoint-specific boxplots respectively. **(D)** Integrative analysis of gene expression and footprint of TFs in SC2 and SC7. The bar plot showing the mean TPM of TF genes and the black bar showing the DEGs. The left and right panels of heatmap showing the expression profiles and footprint score of TFs across the four stages, respectively. The MA plots showing the chromatin accessibility changes (from D0 to D15) of target regions of corresponding TFs, with annotation of the number of increased or decreased peaks. **(E)** Integrative analysis of gene expression and footprint of TFs in SC1, SC4, SC5, SC6, and SC3. The bar plot showing the mean TPM of TF genes and the black bar showing the DEGs. The left and right panel of heatmap showing the expression profiles and footprint score of TFs across the four stages.

TFs often recruit RNA polymerases to DNA sequences to promote gene transcription while chromatin accessibility is important for TF binding (Kagey et al., 2010). The TF binding motif enrichment conducted by HOMER infers potential TFs by comparing the peak sequences with a background set of DNA sequences, which does not provide TF binding sites and the measurement of TF activities. To identify and quantify potential TFs regulating BAT whitening, we performed genome-wide footprinting analysis for TFs, which provides evidence of direct occupancy of TF on genomic DNA. Our results showed footprint score (measured by both the chromatin accessibility and the deleted ATAC-seq signal duo to protein binding) of genes were positively correlated with the expression of key adipose genes. For instance, the BAT-selective gene *UCP1* contained five distinct TF binding regions and all of them showed decreased footprint score from D0 to Y2. Based on the binding motifs, a total of 78 TFs were predicted to interact with the corresponding regions of *UCP1* (Figure 4A). As an upregulated master WAT-selective gene, *LEP* contained two distinct binding regions residing in the upstream regions and both of them showed increased footprint score from D0 to Y2. A total of 27 TFs were predicted to interact with DNA sequences of these regions of *LEP* (Figure 4B). As a master lipogenesis gene, *FABP4* contained four distinct binding regions and all of them showed temporarily decreased footprint score from D0 to D15 and increased footprint score from D15 to Y2. A total of 57 TFs were predicted to interact with these regions of *FABP4* (Supplementary Figure S3A). Thus, we obtained the potential binding sites of subjected TFs in the genome-wide ATAC-seq peaks (Supplementary Table S7).

Based on the genome-wide footprint of TFs, a total of 765 vertebrate TFs were classified into co-active groups (SC1–SC7) across stages, which reflected the BAT whitening and adipose tissue remolding of BAT (Figure 4C). The SC2 and SC7 (n = 87 + 141) displayed highest activity at D0 and then decreased thereafter, suggesting TFs within these clusters are likely involved in maintaining the thermogenic phenotype of BAT. The TFs in SC2 and SC7 overlapped with many known key thermogenic TFs, such as the EBF1 (Angueira et al., 2020), EBF2 (Stine et al., 2016), NFIA (Hiraike et al., 2017), RORA (Auclair et al., 2021), ESSRG (Müller et al., 2020) and THRA (Bertrand-Gaday et al., 2016), explaining their activity at the beginning of thermogenic adaptation in the extrauterine low-temperature environment. As an example, the aggregated decreased footprint signals of EBF2 on the genome were shown in Supplementary Figure S3B. The footprint signals of TFs in SC1 (*n* = 160) displayed the highest activation at D15 and were decreased in D85 and Y2. The SC1 harbored well-known adipogenesis-TF PPARG (Tang and Lane, 2012) and adipose metabolism-TF ESR1 (Zhou et al., 2020). On the other hand, the WAT-selective TF HAND2 was also classified into SC1 (Giroud et al., 2021). As an example, the corresponding pattern of aggregated footprint signals of ESR1 on the genome was shown in Supplementary Figure S3B. The TFs in SC4, SC5, and SC6 (n = 74 + 26 + 116) displayed a particularly prominent D85-selective footprint. Many TFs in these clusters were involved in the adipose stem cell differentiation and adipose tissue expansion, such as CEBPA, CEBPB (Gregoire et al., 1998), PBX1 (Monteiro et al., 2011), and CTCF (Chen et al., 2021), MAFB (Pettersson et al., 2015), which reflected the key stage for adipose tissue remodeling at D85. As an example, the aggregated footprint signals of CEBPB on the genome were shown in Supplementary Figure S3B. SC3 cluster (n = 161) displayed the highest footprint score at Y2, suggesting that TFs in the cluster are likely involved in inhibiting BAT thermogenic phenotype and promoting the final formation of WAT-like phenotype. The TFs in SC3 overlapped with many TFs inhibiting BAT thermogenesis, such as The USF1 (Laurila et al., 2016) TFE3 (Fujimoto et al., 2013), and EGR1 (Milet et al., 2017) and many TFs promoting WAT development, such as CREB1 (Yao et al., 2020), SP1 (Roy et al., 2017), FOSL2 (Wrann and Rosen, 2012), KLF5 (Oishi et al., 2005), ATF1 (Fox et al., 2006). As an example, the aggregated footprint signals of CREB1 on the genome were shown in Supplementary Figure S3B.

Paired comparisons of the four stages identified 5, 27, 27, 25, 26, and 20 TFs significantly increased and 17, 18, 25, 26, 26, and 26 TFs significantly decreased footprint in D15 *vs.* D0, D85 *vs.* D0, Y2 *vs.* D0, D85 *vs.* D15, Y2 *vs.* D15, and Y2 *vs.* D85, respectively (Supplementary Figure S3C). As a most maximum interval, the Y2 *vs.* D0 formed an outstanding group containing TFs with the highest differences in footprint binding scores, including FOS, FOSl1, FOSL2, ATF3, BATF, BACH1, BACH2, NFE2, JDP2, JUN, and BNC2 (Supplementary Figure S3D). By integrating differential binding TFs (DBTFs) and their gene expression, we asked whether the activities of TF were consistent with gene expression themselves during the whitening process of BAT. In SC2 and SC7, we found that most known thermogenic TFs were maintained or increased gene expression during the whole whitening process, while their footprints dramatically decreased after D0, such as RORA, EBF1, NFIA, ESSRG, and EBF2 (Figure 4D). Furthermore, our analysis showed a broadly decrease in chromatin accessibility of regions that were bound by these TFs, including 74.02% of the bound peaks of EBF1, 74.59% of the bound peaks of EBF2, 71.4% of the bound peaks of ESSRG, 72.14% of the bound peaks of NFIA, and 70.75% of bound peaks of RORA (Figure 4D). These results indicated that the rapidly closing chromatin of TF-bound regions, rather than downregulating TF gene expression, might be an important regulation for the loss of BAT thermogenic phenotype in the early thermogenic adaptation period in rabbits. Prediction of protein-protein interaction (PPI) of TFs in SC2 and SC7 found that 13 TFs might form a large complex, which contained EBF1 (Supplementary Figure S4).

We next analyzed TF with an increased footprint in the local or long-term periods. The TFs in SC1, SC4, SC5, and SC6 revealed a concordant pace between TF gene expression and TF footprint during the local period before they reaching to the highest footprint signals. In the group SC1, the upregulated TF gene expression and increased TF footprint were found from D0 to D15, which suggested a coordinated pace between TF gene expression and TF footprint. Prediction of PPI of TFs in SC1 found that nine TFs might form two small complexes, among which one complex contained a well-known adipogenesis-TFs, PPARG (Supplementary Figure S4). In the SC4, SC5, and SC6, the TFs with a coordinated pace between TF gene expression and TF footprint were mainly found from D15 to D85. Prediction of PPI of TFs in SC4, SC5, and SC6 found that nine TFs might form three small complexes, of which the members of the well-known lipogenesis TF family CEBPs formed a large complex (Supplementary Figure S4). The maintaining or upregulating of TF gene expression in the local period before the TF footprint reaching the highest signals suggested gene expression is necessary for TF activity. Although gene expression of many TFs in SC1, SC4, SC5, and SC6 were still maintained or upregulated after they reached the highest footprint signals, their footprints were subsequently attenuated thereafter, due to the chromatin closing of TF-bound regions (Figure 4E). In contrast to TFs in the former groups, the increased footprint of most TFs in SC3 showed to be concordant with their gene expression during the whole period from D0 to Y2 (Figure 4E). The concordant pace between TF gene expression and TF footprint in thus long-term period might suggest TFs in SC3 might be involved in the whole whitening process of BAT, rather than regulating in a local period like TFs in SC1, SC4, SC5, and SC6. Prediction of PPI of TFs in SC3 found that 21 TFs formed a large complex, which contained adipocyte differentiation TF KLF5 (Supplementary Figure S4).

In summary, dynamics of TF footprint suggested that the sequential regulation of potential TFs mediated the loss of thermogenic phenotype and the gain of WAT-like phenotype of BAT. Many TFs, such as EBF1, PPARG, CEBPs, and KLF5, might interact with other TFs to form TF complexes to play key roles.

## Discussion

Although tremendous progress has been made to increase thermogenic fat to defend against obesity, the basis of the natural transition from BAT to WAT remains poorly understood. Previous studies have identified WAT-selective (e.g., *LEP*) and BAT-selective genes (e.g., *UCP1*) by comparing differences between WAT and BAT and revealed that the orchestration of the program of WAT-selective and BAT-selective genes drives the progress of BAT “browning” in mice (Bai et al., 2017; Ding et al., 2018). In this study, dynamics of transcriptome and chromatin accessibility were determined during the BAT whitening. Our data showed that the characteristics of BAT, including tissue color, thermogenic gene expression, and WAT-selective genes gradually developed towards WAT during rabbit growth, suggesting a perfect rabbit model for studying BAT whitening. The CIDEA previously was identified as a BAT-selective gene. Fischer and colleagues identified CIDEA functions molecularly as an indirect inhibitor of thermogenesis (Fischer et al., 2017). Our data revealed that CIDEA was an upregulated gene during the loss of thermogenic phenotype, which was consistent with the results conducted by Fischer and colleagues and indicated that CIDEA might play a positive role in BAT whitening in rabbits.

It is well known that transcriptomic dynamics play important roles in different aspects of adipocyte biology in WAT or BAT (Qiang et al., 2012; Kong et al., 2014). Comparisons of different stages during rabbit BAT whitening revealed a great numbers of DEGs were detected in D15 *vs.* D0 and Y2 *vs.* D85, but relatively few DEGs detected in D85 *vs.* D15, which might indicate that gene expression was more important in the early and terminal whitening process than other browning periods. The GO enrichment of D0-specific DEGs (RC6) reflected the loss of thermogenic capacity, while the DEGs that were upregulated from D0 to D85 (RC4) were enriched in mitochondrial functions, which might indicate that the potential of thermogenic capacity was maintained during the whitening process and could be activated when demand for heat generation. The visceral WAT played an important role in immune functions (Kwon et al., 2012). Our results showed that the continuously upregulated DEGs from D0 to Y2 (RC5 and RC2) enriched GP-BP and KEGG analysis were associated with immune response-related terms and pathways (such as lysosome and cytokine-cytokine receptor interaction), which might indicate the gain of immune functions after the loss of thermogenic capacity of BAT. The upregulated DEGs from D0 to Y2 were also enriched in the well-known adipogenesis-related KEGG pathways, such as the TGF-beta signaling pathway (Yadav et al., 2011), MAPK signaling pathway (Anderson, 2006), and calcium signaling pathway (Dai et al., 2021), indicating these genes might be involved in the process of lipid accumulation during BAT whitening.

In this study, we utilized ATAC-seq technology to determine accessible regions of chromatin in rabbit BATs, which is the first comprehensive assessment of chromatin accessibility in rabbit species. Our hierarchical clustering analysis based on the peak intensity showed the high reproduction of replicate samples, which indicated that three replicates can reflect each stage. Based on the genomic annotation of peaks, we profiled the ATAC-seq signal enrichment in the regions around gene bodies and found the enrichment patterns for genes were in line with that reported in previous ATAC-seq studies (Ackermann et al., 2016; Hu et al., 2020), suggesting reliable data in our study. The thyroid hormone signaling pathway plays a positive role in the activation of BAT (Yau et al., 2019). The nearby genes of DPs that increased from D0 to D15 (K4) were strongly enriched in the thyroid hormone signaling pathway, which indicated that chromatin accessibility could regulate thermogenic adaptation of BAT via regulating genes in the pathway. The thermogenesis of BAT was closely associated with many lipid-metabolism processes, such as oxidation of fatty acids, decomposition of fatty acids and citric acid cycle (Paulus et al., 2017; Richard et al., 2020). The lipid metabolism pathways were significantly enriched by the DPs K4, suggesting the open chromatin of genes involved in this pathway was essential for thermogenesis during the early life of rabbits. Interestingly, our clustering analysis showed that DPs increased from D15 to D85 (K6), and were enriched in lipid accumulation-associated pathways, which was similar to the enriched results for the continuously upregulated DEGs from D0 to Y2 (RC5 and RC2). Similarly, biological processes and pathways involved in immunity functions were enriched by both Y2 highly expressed DEGs (RC5) and Y2 highly accessible peaks (K3), which might indicate functional synergy of chromatin accessibility and gene expression at D85 and Y2.

Currently, the methods of peak annotation were based on linear overlapping with known genes (Quinlan, 2014; Yu et al., 2015). The correlation between accessibility and gene expression level decreased with the increasing distance between the accessible peak and the TSS (Wang F. X. et al., 2020). In this study, the integrative analysis of ATAC-seq data and RNA-seq data showed many peaks were not full correlated with the expression of peak-related genes. Here, we present several reasons for this observation: (1) distal intergenic peaks are not necessarily influencing their closest genes and there was high dimensional regulation of gene elements during BAT whitening, such as enhancer regulation, which might need further Hi-C data to infer. (2) one gene could be regulated by multiple TFs, while the abundance and transcriptional effect of different TFs make it complicated for gene expression. (3) the current annotation of the rabbit genome is not comprehensive enough, for instance, the high proportion of novel long non-coding RNAs of rabbits were found mainly residing in the intergenic regions, which might cause the bias of peak annotation (Wang et al., 2018). Nevertheless, we found a high correlation between chromatin accessibility and gene expression in the key adipose genes, which indicated that chromatin accessibility was important for fat biology. On the other hand, the positive correlation between chromatin accessibility and gene expression in the period from D85 to Y2 was higher than that in the period from D0 to D15, which might suggest the regulation effect of chromatin accessibility was increased with the process of BAT whitening in rabbits.

It is well known that TFs regulate gene expression programs in cell commitment and tissue development through binding specific motifs on the genome, and the architecture of accessible chromatin provides an opportunity to detect TF binding events (Kagey et al., 2010; Buenrostro et al., 2013). EBF1 was previously considered a TF involved in white adipocyte differentiation (Gao et al., 2014). A recent study revealed that deletion of EBF1 alone in adipocytes had minor effects on BAT (Angueira et al., 2020). Our data predicted that EBF1 was involved in a large TF complex, which indicated that EBF1 might maintain the thermogenic phenotype of BAT by interacting with other TFs in rabbits. PPARG is a TF that is essential for both BAT and WAT (Picard et al., 2004; Seale et al., 2008). Both gene expression and footprint of PPARG increased from D0 to D15, and PPARG was predicted to be involved in a TF complex, which suggested that PPARG might play an important role in regulating BAT development and organizing the TF network during the early thermogenic adaptation period. A previous study revealed that DNL was an important biological process in the transformation of brown, beige, and white adipose tissues (Mottillo et al., 2014). Our gene expression data showed DNL-related genes were highly expressed at D85. On the other hand, the TF footprinting analysis showed that many TFs with high footprint signals in D85 were involved in the adipose stem cell differentiation and adipose tissue expansion, such as CEBPA, CEBPB, PBX1, CTCF, and MAFB. On the other hand, our data showed that the members of the CEBPs family might form TF complex, which indicated that D85 is a vital stage for the lipid synthesis of rabbit BAT and CEBPs might be crucial for *NDL* of BAT in rabbits. The combined analysis of TF gene expression and TF footprint showed the decreasing chromatin accessibility of target genes contributes to the loss of the thermogenic phenotype of BAT during the early thermogenic adaptation. Except for the classical thermogenic BAT TFs, many TFs in SC2 and SC7 warrant further investigation. The gene expression of many TFs in SC1, SC4, SC5, and SC6 were maintained or upregulated after TF reaching the highest footprint, which indicated that gene expression of these TFs is necessary for BAT, while their activities were also affected by chromatin accessibility of their binding regions. Therefore, chromatin accessibility plays a vital role in epigenetic regulation during BAT whitening in rabbits. KLF5 was previously reported to play a key role in 3T3-L1 differentiation and its expression was induced by C/EBPB and C/EBPD (Oishi et al., 2005). Our result showed that KLF5 was highly expressed and maintained a high footprint followed the CEBPs activation (D85) in Y2 samples, which might suggest local sequential TF-activation from D85 to Y2 and KLF5 was potential TF contributing to regulating the whitening process of BAT in elder rabbits.

## Conclusion

In summary, our study provided the transcriptional and epigenetic frameworks for understanding BAT whitening in rabbits for the first time and identified potential TFs involved in BAT whitening, which facilitated potential insights into fighting against obesity.

## Data Availability

The datasets generated for this study can be found in the Sequence Read Archive (https://www.ncbi.nlm.nih.gov/sra) at NCBI, with the BioProject ID: PRJNA716375.
