# Supplementary material for: Dynamics of transcriptome and chromatin accessibility revealed sequential regulation of potential transcription factors during the brown adipose tissue whitening in rabbits
Source: Front Cell Dev Biol. 2022 Sep 26;10:981661. doi: 10.3389/fcell.2022.981661 (PMC9548568; doi:10.3389/fcell.2022.981661)
Supplement: Supplementary file 2 [file Table1.DOCX]

**Table S1 Primers of genes used in RT-qPCR**

| **Gene ID** | **Ensembl ID** | **Primer sequences (forward/reverse)** | **Product length (bp)** |
| --- | --- | --- | --- |
| *QARS1* | ENSOCUG00000005082 | CATTGAACACATCACCCACTCAC/CCTCTTGGAGACAACAGCATAGT | 151 |
| *VPS29* | ENSOCUG00000011924 | ACTGGGAACCTTTGCACCAA/TCGAAGTCCCCTCTCACGAT | 83 |
| *TCF25* | ENSOCUG00000013600 | GATGCGGAAATAAGTCAGCCG/TCCAGAGGAAATGTGACCGC | 76 |
| *ANK2* | ENSOCUG00000010618 | AACTGGGATCAGAAGGCAGC/GAGCTCTTCACTGCTAAGCTCT | 222 |
| *CCDC80* | ENSOCUG00000023764 | CTGTGTCAGCACAAGGGACT/AGCTTTCAGAGTACCGCCAC | 208 |
| *ARHGDIB* | ENSOCUG00000006010 | TGGGGTTCTTTGTGGGACAC/GGTCTCAGGCAGAACATAATAGT | 80 |
| *FABP4* | ENSOCUG00000007445 | GGCCAGGAATTTGATGAAGTC/AGTTTATCGCCCTCCCGTT | 140 |
| *LEP* | ENSOCUG00000010189 | GCGGAAAGTCCAGGATGACA/TGGATCACATTTCGGGACGG | 216 |
| *GSN* | ENSOCUG00000013676 | AGCGCTACATTGAGACGGAC/GTCAAACCACCCGAGGCTAA | 227 |
| *ZC3H12A* | ENSOCUG00000010856 | CAGGCAAGGACAAAAGTGCC/AGTTCACCTTCTGTGGGCAG | 73 |
| *CIDEA* | ENSOCUG00000011380 | TAGGGGACAACACGCACTTC/CTCTGGCGATTCCCGATCTC | 105 |
| *TGFBI* | ENSOCUG00000004928 | TTTTGCAAAGCTCTTGCCCG/CCATCCACACAGGCGTACAT | 230 |
| *BNC2* | ENSOCUG00000004460 | ACATGGCAAAACGCTGACAC/CAAAAGTCTGCACCCAGCAC | 92 |
| *SNCG* | ENSOCUG00000013292 | ACATTGTGGTCACCTCTGGG/TACTTTCCCCCACTCTTGGC | 121 |
| *YIPF3* | ENSOCUG00000005499 | TCCTCTCTCTCCCATTGTCGT/CTTCAGCCCGCCTAAATCAGA | 185 |
| *HADH* | ENSOCUG00000014271 | ATTGTGAACCGTCTGCTGGT/CTGCCGTCGCTGTAGAAAGA | 247 |
| *GMPS* | ENSOCUG00000001480 | CAGGTACTTGCAGGATTCCCA/AAGTAATCACCGCAGAGGCG | 72 |
| *UCP1* | ENSOCUG00000002297 | TGGGCACACTCGCATATTGT/TCGCTGGATTTTGCACAACG | 96 |
| *PPARGC1A* | ENSOCUG00000014668 | AAAAGCTTGACTGGCGTCAC/ACTGCACCACTTGAGTCCAC | 202 |
| *ND1* | ENSOCUG00000029086 | ACCCTAGCAGAAACCAACCG/TCCACATTGAAGCCGGAGAC | 77 |
| *ND2* | ENSOCUG00000029090 | AGGAATAGCCCCCTTCCACT/CCACCTCAACCGCCAACTAT | 189 |
| *COX2* | ENSOCUG00000029099 | TCCGCATGCTAATCTCCTCG/TCCGGGAATGGCATCTGTTT | 83 |
| *CYTB* | ENSOCUG00000029115 | ATGAATCGGAGGCCAACCAG/TTCGATTAGGCTTGCGAGGG | 112 |
| *COX1* | ENSOCUG00000029096 | TACCCCGACGGTACTCAGAC/TTGAGGCGAAGGCTTCTCAG | 129 |
| *SGK1* | ENSOCUG00000014367 | TTTCCTACCGCAGAGCGTTT/TGGGATCAAGCATACCACACT | 88 |
| *RN18S* | ENSOCUG00000027166 | ATCAGATACCGTCGTAGTTC/TTCCGTCAATTCCTTTAAG | 167 |
