# Supplementary material for: Dynamics of transcriptome and chromatin accessibility revealed sequential regulation of potential transcription factors during the brown adipose tissue whitening in rabbits
Source: Front Cell Dev Biol. 2022 Sep 26;10:981661. doi: 10.3389/fcell.2022.981661 (PMC9548568; doi:10.3389/fcell.2022.981661)
Supplement: Supplementary file 6 [file Table3.DOCX]

**Table S3** **Summary of ATAC-seq data and reads mapping**

| Sample | Clean reads | Clean reads Q20 (read1/read2) | Clean reads Q30 (read1/read2) | Total mapped reads | Uniquely mapped reads | FRiP |
| --- | --- | --- | --- | --- | --- | --- |
| D0_1 | 255946708 | 97.64%/97.99% | 93.51%/94.30% | 217797202 (85.09%) | 142881766 (55.82%) | 0.085 |
| D0_2 | 280497902 | 95.86%/98.25% | 88.87%/94.88% | 259223989 (92.42%) | 174724622 (62.29%) | 0.077 |
| D0_3 | 256239784 | 97.60%/97.94% | 93.45%/94.23% | 233166811 (91.00%) | 153888919 (60.06%) | 0.080 |
| D15_1 | 261970772 | 98.24%/98.12% | 95.08%/94.62% | 241774162 (92.29%) | 163901667 (62.56%) | 0.060 |
| D15_2 | 269127628 | 98.21%/98.13% | 95.02%/94.64% | 249076010 (92.55%) | 171721173 (63.81%) | 0.070 |
| D15_3 | 265740496 | 98.24%/98.14% | 95.09%/94.68% | 243674612 (91.70%) | 165673214 (62.34%) | 0.075 |
| D85_1 | 254217054 | 98.17%/97.71% | 94.90%/93.72% | 214945999 (84.55%) | 154163662 (60.64%) | 0.102 |
| D85_2 | 243539454 | 98.06%/97.80% | 94.58%/93.94% | 212993453 (87.46%) | 152256489 (62.52%) | 0.124 |
| D85_3 | 234159034 | 98.16%/97.64% | 94.94%/93.60% | 209249361 (89.36%) | 149874649 (64.01%) | 0.108 |
| Y2_1 | 226067992 | 98.16%/97.94% | 94.87%/94.25% | 186428690 (82.47%) | 137736207 (60.93%) | 0.123 |
| Y2_2 | 225657996 | 98.19%/97.87% | 94.94%/94.10% | 180885323 (80.16%) | 134072056 (59.41%) | 0.088 |
| Y2_3 | 226849584 | 98.16%/97.91% | 94.85%/94.17% | 193720071 (85.40%) | 144591479 (63.74%) | 0.077 |
