# Supplementary material for: Dynamics of transcriptome and chromatin accessibility revealed sequential regulation of potential transcription factors during the brown adipose tissue whitening in rabbits
Source: Front Cell Dev Biol. 2022 Sep 26;10:981661. doi: 10.3389/fcell.2022.981661 (PMC9548568; doi:10.3389/fcell.2022.981661)
Supplement: Supplementary file 7 [file DataSheet2.docx]

Supplementary Material

# Supplementary Tables

## Table S1 Primers of genes used in qRT-PCR (docx format).

## Table S2 Differential analysis of RNA-seq (xlsx format).

## Table S3 Summary of ATAC-seq data and reads mapping (docx format).

## Table S4 Genome-wide identified ATAC-seq peaks (xlsx format).

## Table S5 Significantly enriched TF binding motifs (xlsx format).

## Table S6 Combined analysis of chromatin accessibility and gene expression based on peak annotation (xlsx format).

## Table S7 The potential binding sites of subjected TFs in the genome-wide ATAC-seq peaks (zip format).

# Supplementary Figures

**
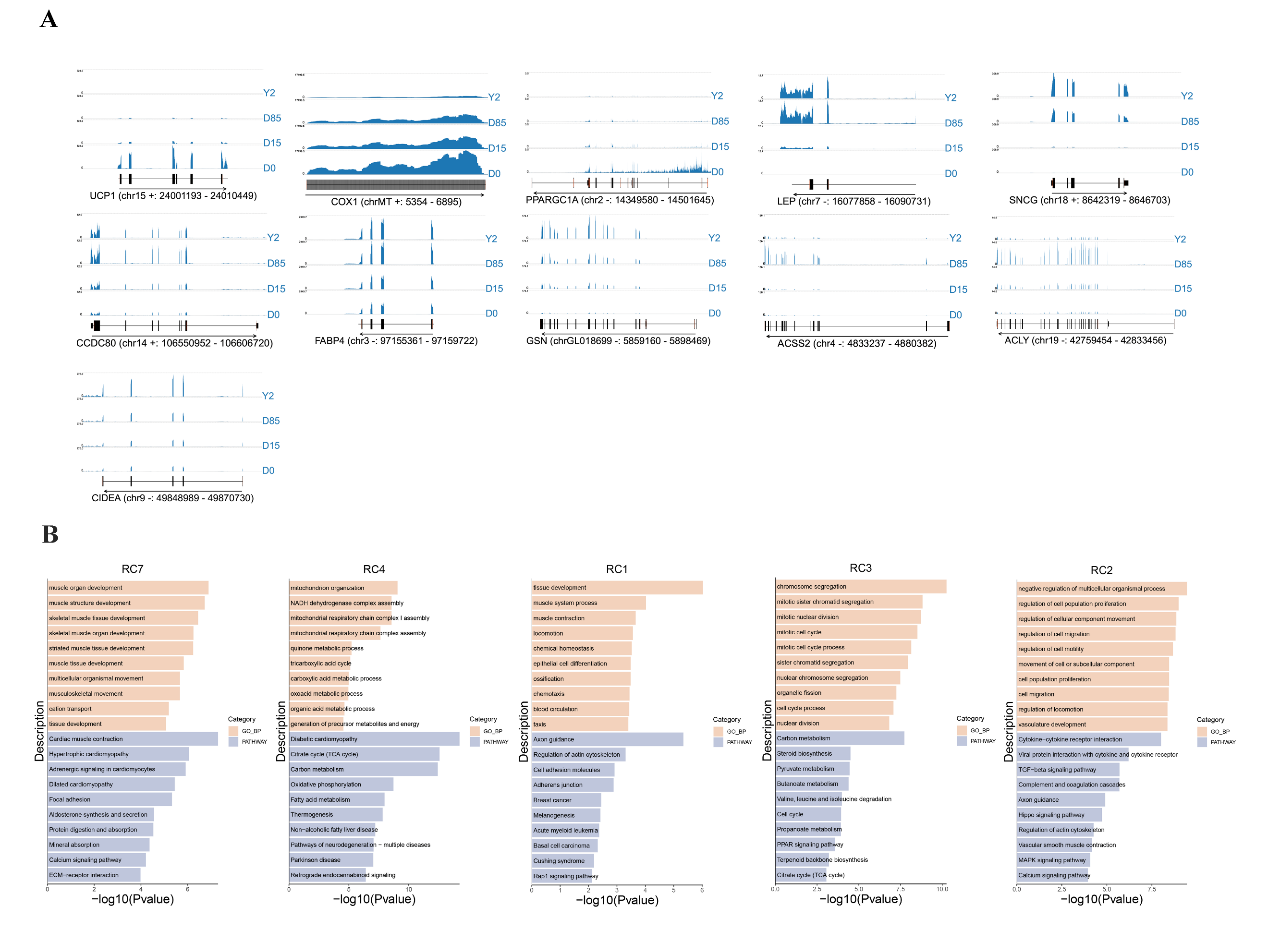
**

**Figure S1. The genomic tracks of key genes as well as the functional and pathway enrichment for RNA-seq.** (A) Genomic tracks for key genes. Sequencing reads were normalized using TPM. The wide genomic elements represent the CDSs, the narrow genomic elements represent the UTRs, and the red genomic elements represent the start or end code of genes. (B) Top 10 GO-BP terms and top 10 KEGG pathways enriched by genes in corresponding cluster.


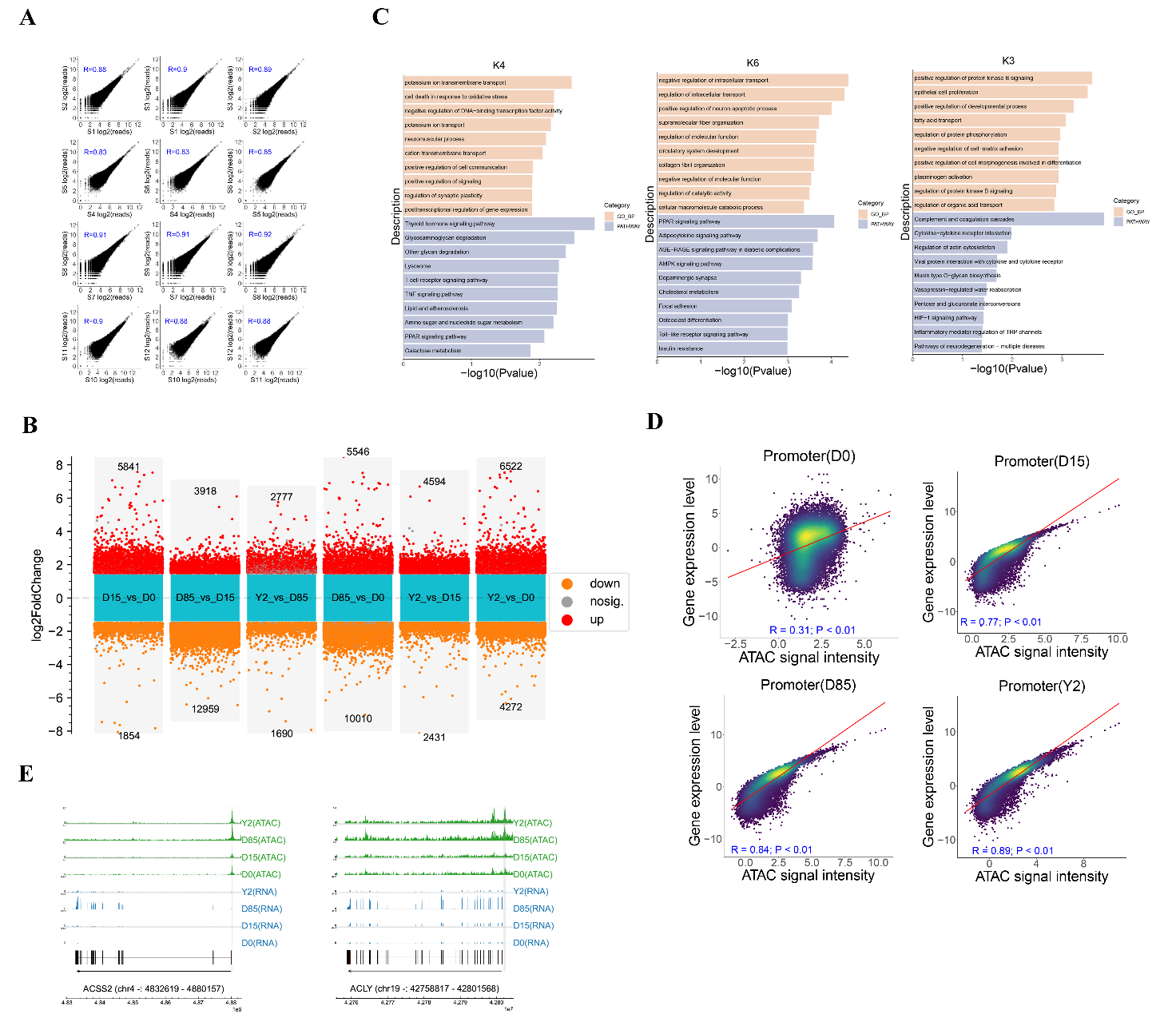


**Figure S2. Landscape and dynamics of chromatin accessibility of rabbit BATs.** (A) Correlation analyses of the ATAC-seq libraries. Pearson coefficients were based on raw counts of peaks. S1-S3 represents samples in D0, S4-S6 represents samples in D15, S7-S9 represents samples in D85, and S10-S12 represents samples in Y2. (B) Differential analysis of peaks. The scatters showing the peaks with log2(FC) > 1.5 or log2(FC) < －1.5. The red and orange scatters showing the peaks with FDR < 0.01. The grey scatters showing the peaks with FDR ≥ 0.01. (C) GO enrichment and KEGG pathways analysis of cluster K4, K6, and K3. (D) The correlation coefficients (Pearson’s correlation coefficients) between intensity of chromatin accessibility of promoters (CPM values) and gene expression levels (TPM values). (E) Tracks of two *DNL* genes.


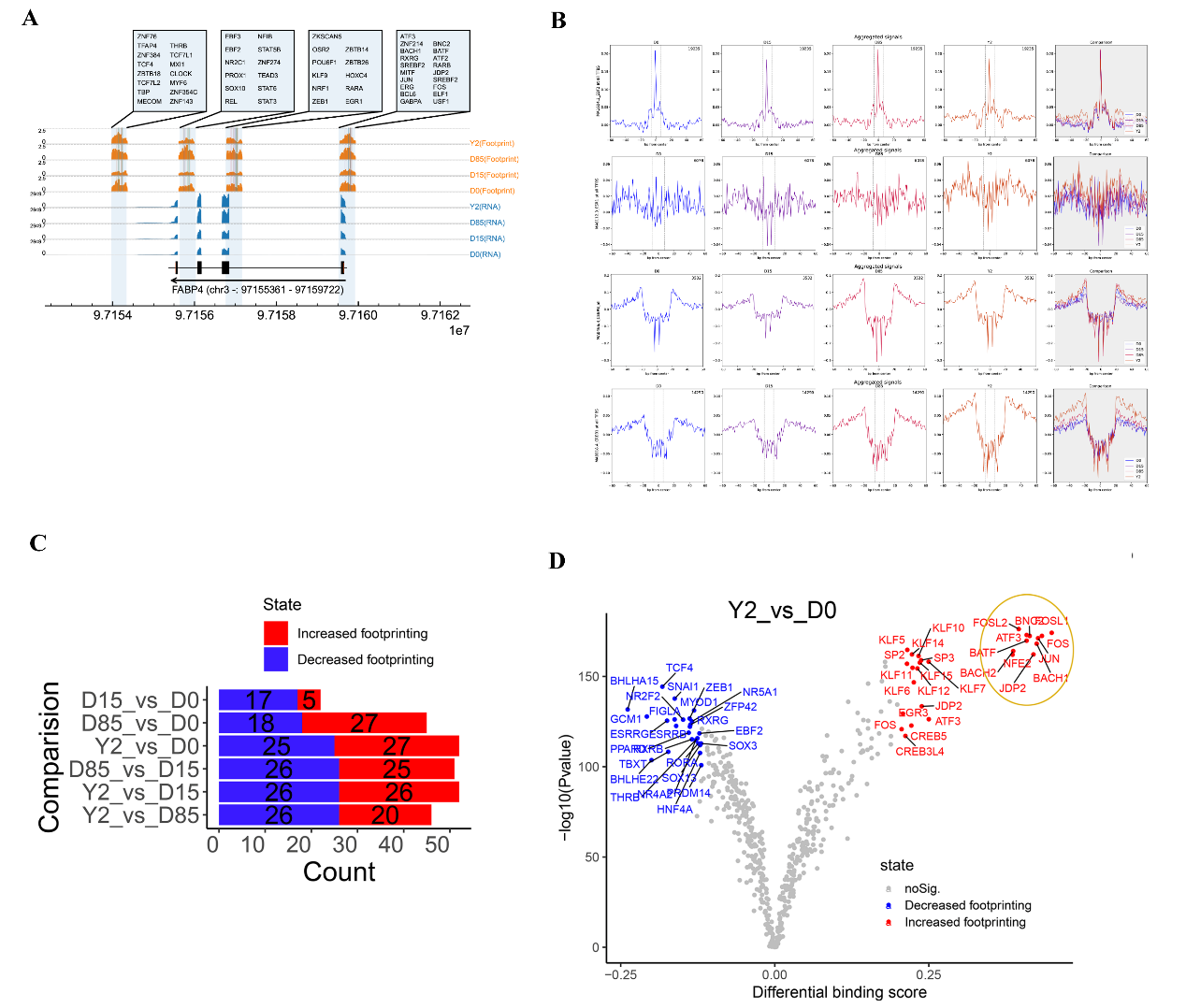


**Figure S3. TF footprinting analysis.** (A) The TF footprints at the loci of FABP4. (B) Instances of aggregated footprint signals of representative TF in corresponding clusters.(C) Barplot of number of TFs have differential footprint. (D) Volcano plot showing the differential binding activity against the −log10(p-value) of all investigated TFs in Y2 *vs.* D0. Each dot represents one TF. A prominent TF group is highlighted by an elliptical frame.


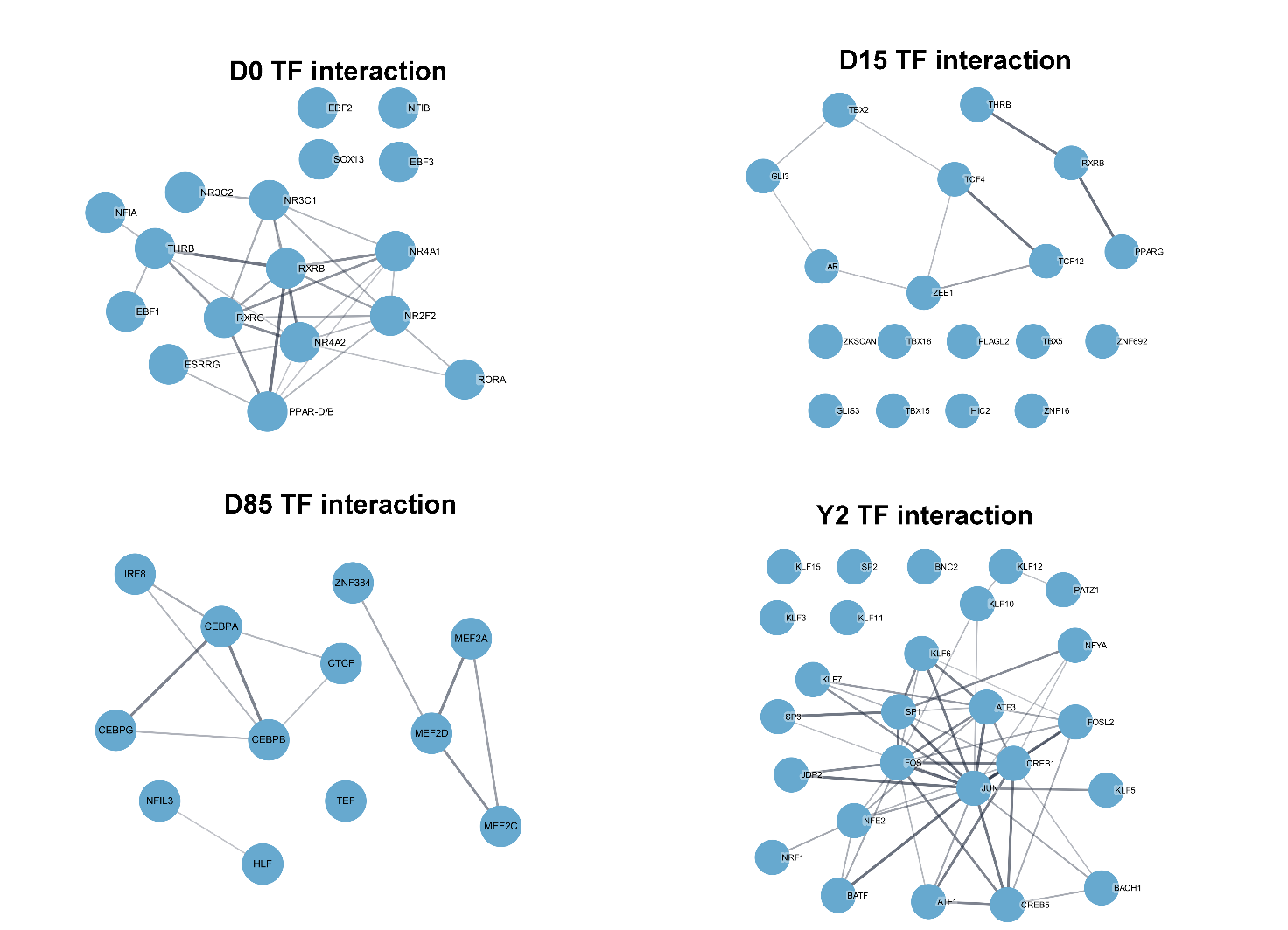


**Figure S4. TF complex analysis.** The networks show the predicted protein-protein interactions at corresponding whitening stage of BATs in rabbits. The size of edges presents the interaction score in String database.
